# Transoral removal of a hilo‐parenchymal submandibular sialolith

**DOI:** 10.1002/ccr3.5903

**Published:** 2022-05-27

**Authors:** Yo‐Hei Kataoka, Yoshihiro Kojima, Ruri Ishibashi, Yuji Nakao, Koji Yamamura, Shotaro Takahashi, Takafumi Hashiba, Takahito Matsue

**Affiliations:** ^1^ Self‐Defence Forces Central Hospital Setagaya‐ku Japan

**Keywords:** minimally invasive technique, submandibular sialolith, TASL, transoral approach

## Abstract

In sialolithiasis, the lithiasis is often large and located at the junction of the middle and posterior third of the duct, in the hilum region. In such cases, transoral approach for submandibular lithiases (TASL) is a useful treatment of choice in patients with large submandibular stones that can be palpated bimanually.

## Abstract 2

TASL is an established minimally invasive technique described more than 20 years ago. However, it is important to report these findings in literature, since previous reports suggested that it is an underutilized surgical technique.

## 1 INTRODUCTION

Sialolithiases mainly affect the submandibular gland (SMG). Often, the lithiasis is large and is located at the junction of the middle and posterior third of the duct, in the hilum region. In such cases, proximal stones are generally removed from the SMG by a transcervical submandibular sialoadenectomy.

Recently, a gland‐preserving technique has been introduced for transoral proximal sialolith removal, which is also termed as the transoral approach for submandibular lithiases (TASL).^1^ Herein, we report a case of transoral removal of a hilo‐parenchymal submandibular sialolith by TASL.

## 2 CASE HISTORY

**FIGURE 1:**
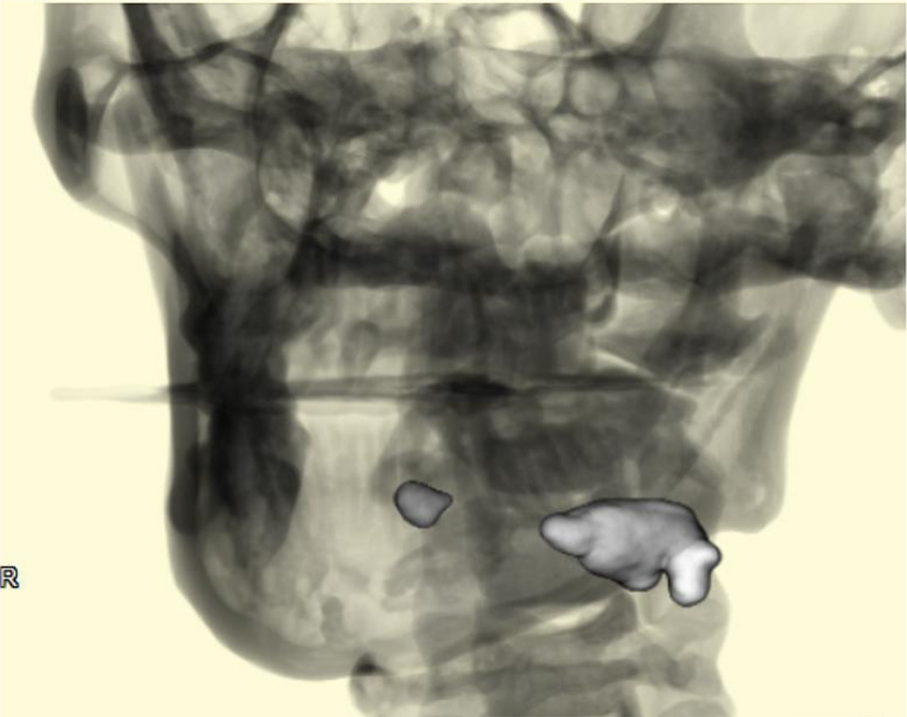
Three‐dimensional cone beam‐computed tomography reconstruction of the left submandibular parenchymal stones

**FIGURE 2:**
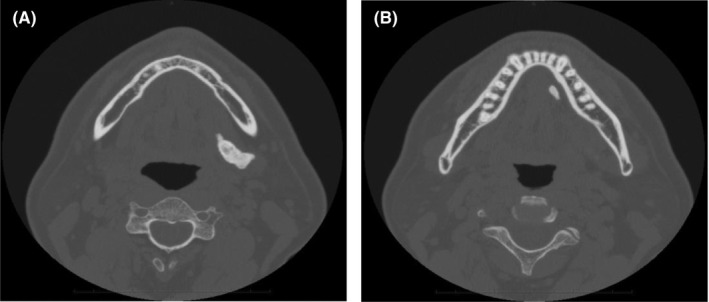
Axial computed tomography images show the left anterior and posterior stones. The posterior (A, 27 mm) and anterior (B, 9.4 mm) stones are visible

A 42‐year‐old man was referred to our hospital for the assessment of an asymptomatic radiopaque lesion in the left submandibular region. Panoramic radiography and computed tomography confirmed two calcified lesions in the posterior and anterior regions of Wharton's duct, respectively (Figures 1 and 2). Intraoral examination by bimanual palpation revealed a small, firm, and nontender swelling in the anterior floor of the mouth and a large, firm, and nontender swelling in the posterior floor. The final diagnosis was sialolithiasis in the left Wharton's duct and hilo‐parenchymal submandibular area.

**FIGURE 3:**
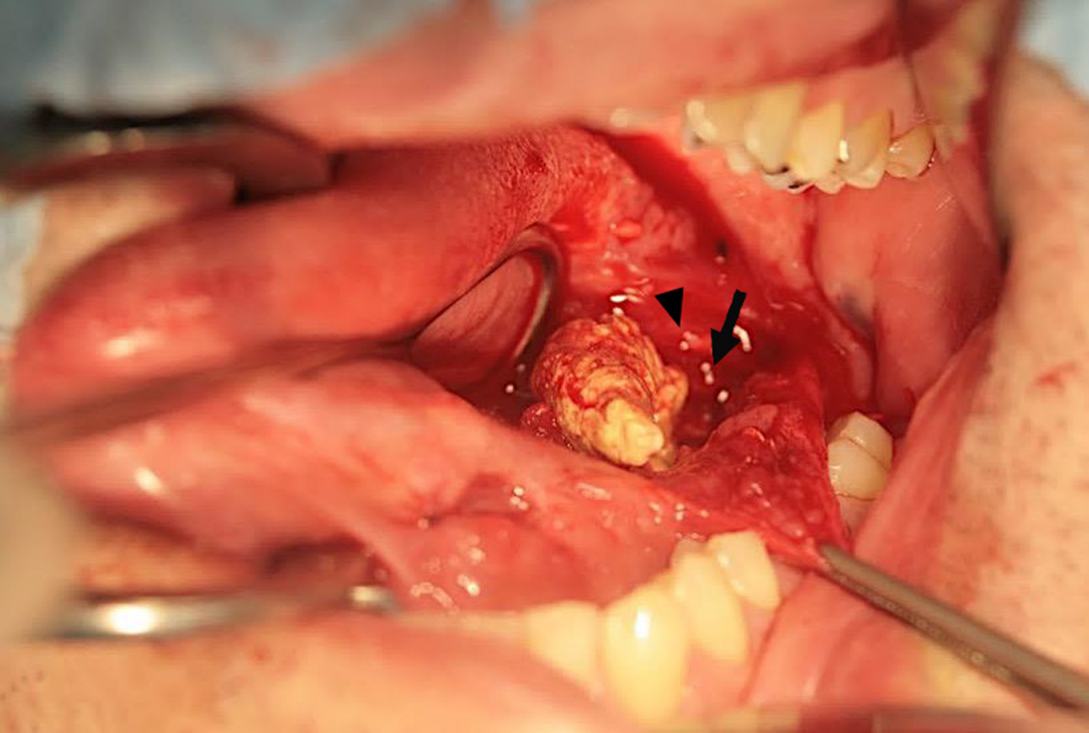
The stone extracted from the parenchyma and its relationship with Wharton's duct (arrow) and the lingual nerve (arrow head)

In the operating room, the patient was placed in the dorsal decubitus position. After transnasal intubation and proper oral preparation, the buccal floor was infiltrated under the mucosa with a saline solution with 2% epinephrine (0.50 mg in 20 cc). An incision was made through the mucosa of the lateral floor of the mouth, from the orifice of Wharton's duct to the lingual side of the retromolar region, leaving a cuff of normal lingual mucosa to facilitate subsequent wound closure. The anterior sialolith was pushed out of the duct and removed via manual manipulation. Careful dissection was performed between Wharton's duct and the lingual nerve. External digital pressure was applied to facilitate the isolation of the duct from the lingual nerve up to the hilum of the SMG. After localizing the posterior stone with bimanual palpation, the duct was incised, and the stone was removed (Figure 3). The duct was then irrigated with normal saline to clean the region and remove stone debris. The incised mucosa at the floor of the mouth was sutured back to its original position, without repairing the incision site of Wharton's duct. The patient's postoperative course was uneventful, with no significant complications. So far, during the postoperative follow‐up period of two years, there has been no evidence of recurrence.

## 3 DISCUSSION

Sialolithiasis is the most common salivary gland pathology. SMG resection is the standard operative procedure used for the management of proximal sialolithiasis. However, the associated incidence of iatrogenic injuries is relatively high. Recently, several conservative and minimally invasive techniques have been developed for salivary lithiasis surgery, with the development of the sialendoscope and lithotripter.^2^, ^3^, ^4^, ^5^, ^6^

However, a sialendoscope alone is incapable of removing larger (>6 mm), fixed, palpable submandibular stones.^7^, ^8^, ^9^ Additionally, it costs a lot for initial investment and maintenance.

The management of SMG lithiases is based on three criteria: the gland involved, topography of the lithiasis, and the diameter of the lithiasis, according to the GTD classification: the gland involved (G), topography of the lithiasis (T), and the diameter of the lithiasis (D).^10^ The transoral approach is recommended for palpable, impacted, large lithiases (diameter >8 mm) situated in the posterior third of Wharton's duct. Using the GTD classification, lithiases classified as submandibular lithiases over 8 mm in diameter (large and impacted) and situated in the posterior third of Wharton's duct are better operated with TASL. This surgical procedure is minimally invasive, repeatable, allows functional recovery of the gland after obstruction removal, and minimizes scarring,^1^ even for large lithiases. However, previous reports have suggested that TASL is an underutilized surgical technique.

McGurk et al.^11^ reported that small stones that cannot be palpated are a contraindication for intraoral removal. In their patient cohort, they observed that stones that were palpable on bimanual examination tended to be easier to retrieve; this was attributed to the fact that nonpalpable stones reside in the gland and their position is masked by the surrounding tissues. Intraoral dissection is rarely performed when the stone is severely adherent to the surrounding tissues, as the approach to the transcervical route may be altered. Thus, appropriate preoperative assessment via manual palpation is important in the context of informed consent.

Our case highlights the possibility that intraoral removal of proximal submandibular stones with the preservation of the gland and ductal system is safe. The results in our patient seem to confirm this surgical choice, but further cases and larger‐scale studies are needed to validate this association as a valid option for traditional transcervical surgery.

## 4 CONCLUSIONS

There is a possibility that TASL is considered as the treatment of choice in patients with large submandibular stones that can be palpated bimanually.

## AUTHOR CONTRIBUTIONS

Yo‐hei Kataoka, Yoshihiro Kojima, Ruri Ishibashi, Yuji Nakao, Koji Yamamura, Shotaro Takahashi, Takafumi Hashiba, and Takahito Matsue. YK, RI, YN, KY, and ST: gathered the patient data, performed a literature review, and wrote the manuscript. TH: reviewed, corrected patient data, and revised the manuscript. TM: was involved in overall supervision of the paper. All authors: read and approved the final manuscript.

## CONFLICTS OF INTEREST

The authors declare that they have no conflict of interest.

## CONSENT

Written informed consent was obtained from the patient to publish this report in accordance with the journal's patient consent policy.
